# Identification of microRNA biomarkers in atrial fibrillation: A protocol for systematic review and bioinformatics analysis

**DOI:** 10.1097/MD.0000000000016538

**Published:** 2019-07-26

**Authors:** Nan-Nan Shen, Zai-Li Zhang, Zheng Li, Chi Zhang, Hao Li, Jia-Liang Wang, Jun Wang, Zhi-Chun Gu

**Affiliations:** a Department of Pharmacy; b Department of Geriatrics, Affiliated Hospital of Shaoxing University, Shao Xing, Zhejiang Province; c Department of Pharmacy; d Department of Cardiology, Renji Hospital; e Department of Pharmacy, Shanghai Children's Medical Center, School of Medicine, Shanghai Jiaotong University, Shanghai, China.

**Keywords:** atrial fibrillation, bioinformatics analysis, meta-analysis, microRNA

## Abstract

**Background:**

Atrial fibrillation (AF) is recognized as the most prevalent arrhythmia, and its subsequently serious complications of heart failure and thromboembolism always raise the social attention. To date, the molecular pathogenesis of AF has largely remained unclear. Publications of contemporary studies have evaluated individual miRNAs expression signatures for AF, and findings of different studies are inconsistent and not all miRNAs reported are actually important in the pathogenesis of AF.

**Methods:**

Medline, Embase, and Cochrane Library databases will be comprehensively searched (up to April 30, 2019) for studies identifying miRNA expression profiling in subjects with and without AF. Log10 odds ratios (logORs) and associated 95% confidence interval (95%CI) will be calculated using random-effects models. Subgroup analysis will be performed according to miRNA detecting methods, species, sample types, and ethnicities. Sensitivity analysis will be conducted to detect the robustness of the findings. The methodological quality of studies will be independently assessed using criteria adopted from the Quality Assessment of Diagnostic Accuracy Studies (QUADAS-2). Furthermore, bioinformatics analysis will be performed to identify the potential target genes in AF and the corresponding pathways of dysregulated miRNAs. Two reviewers will independently screen potential studies and extract data in a structured eligibility items, with any disagreements being resolved by consensus.

**Results:**

The present systematic review will identify potential biomarkers by pooling all differentially expressed miRNAs in AF studies, as well as to predict miRNA-target interactions and to identify the potential biometric functions using bioinformatics analysis.

**Conclusions:**

This systematic review and bioinformatics analysis will identify several miRNAs as potential biomarkers for AF, and explore the biological pathways regulated by the eligible miRNAs.

**PROSPERO registration number:**

CRD42019127594

## 1 Introduction

Atrial fibrillation (AF) is the most frequent arrhythmia worldwide, which is detected in a third of all ischemic strokes and is responsible for most of fatal strokes, causing a substantial socioeconomic burden.^[1]^ With aging populations, the prevalence of AF has been significantly increased.^[2,3]^ The number of AF patients was predicted to approach 6 to 12 million in the United States by 2050 and 17.9 million in Europe by 2060.^[4]^ Regretfully, the definite molecular mechanisms underlying AF pathogenesis are complex and poorly understood.^[5]^ For AF patients, pharmacological approaches currently available are lack of sufficient efficacy, and the management is just focus on reducing symptoms and preventing complications. In the last decade, a number of studies tried to find out the transcriptomic changes in both AF patients and animal models using microarray technologies.^[6–9]^ And several key pathways related to microRNAs (miRNAs), such as Ca^2+^-dependent signaling pathways, inflammatory and immune pathways, apoptotic and cycle pathways have been found,^[10]^ which indicates that miRNAs are likely to be the therapeutic target for AF.^[11]^

miRNAs, a class of conserved non-coding molecules of 18 to 25 nucleotides, are involved in repressing the expression of mRNA that targets at the post-transcriptional level.^[12]^ miRNAs play critical role in cell differentiation, proliferation, and survival by binding to complementary target mRNAs, resulting in mRNA translational inhibition or degradation.^[13]^ As a pivotal regulator of gene expression, miRNAs are found to be dysregulated in multiple diseases including cancer, neurological, and cardiovascular diseases.^[14–16]^ The differently altered miRNAs in disease conditions may provide a novel therapeutic target for the treatment of diseases.^[17,18]^ In recent years, prominent roles for miRNAs have been revealed in several cardiovascular diseases including cardiac hypertrophy and fibrosis.^[19,20]^ Reduced miR-29 expression was found in the atrial of dogs with heart failure,^[21]^ and delivery of antagomiR-29 in a mouse model resulted in a significant reversal of fibrosis.^[22]^ Another miRNA, miR-208, is a cardiac-specific and important in cardiac hypertrophic response, and its expression is sufficient to induce arrhythmias in mice.^[23,24]^

Currently evidence is accumulated for a clinically relevant pathophysiological role of miRNA in AF. Aberrant miRNA expression could be observed in both animal models and AF patients in both cardiac tissue and blood.^[25]^ It is frequently that miRNAs expression differences exist in patients with AF. For example, compared with patients without AF, miR-638 and miR-486 are upregulated, while miR-572 and miR-423 are downregulated in myocardium of AF patients.^[9,26–29]^ miR-99b expression level is down-regulated both in plasma^[4,30,31]^ and left atrial appendages (LAA),^[32]^ while miR-328 is reported to be decreased in plasma,^[4]^ and increased in atrial tissue.^[33]^ In addition, the expression pattern of AF-associated miRNA differs among tissues. One study revealed that 65 and 42 AF-associated miRNAs were found significantly dysregulated in right atrial appendages (RAA) and left atrial appendages (LAA) of AF patients respectively. And 23 of them were found both in RAA and LAA, 45 of them were found only in RAA, while 19 of them were found only in LAA.^[34]^ Moreover, miRNAs are also involved in AF-related remodeling processes and have critical roles in the pathways of AF pathogenesis.^[25]^ Based on controversial conclusions in previous studies, a systematic review and meta-analysis is necessary to identify and unify miRNAs expression signatures in AF. Finally, specific miRNA may be candidate for potential biomarkers and new therapy target for AF.^[19,35]^

MiRNAs regulate relevant target genes and play vital roles in the development of AF. Multiple explorative studies have uncovered that miRNAs are involved in atrial electric remodeling in AF by targeting critical genes. For example, miR-328 contributes to the adverse atrial electric remodeling by targeting CACNA1C and CACNB1 genes,^[33]^ and miR-21 inhibits cardiac fibrosis via upregulating WWP-1 in the TGF-β1/Smad2 signaling pathway.^[36]^

Emerging evidence suggests that a large number of differentially expressed miRNAs are validated to regulate relevant target genes and play vital roles in AF.^[10,37]^ However, findings of different studies are inconsistent and not all miRNAs reported are actually important in the pathogenesis of AF. This systematic review will focus on miRNAs expression signatures and predict miRNA-target interactions using bioinformatics analysis in current available AF studies. We aim to determine the value of miRNAs biomarkers for AF and provide a better understanding for biological characteristics of miRNAs in AF.

## 2 Methods

### 2.1 Data sources and search strategy

The literature search will be conducted in databases of Medline, Embase, and Cochrane Library from inception to April 30, 2019 using the following terms: (miRNA OR microRNA OR miR-) AND (atrial fibrillation OR AF) AND (expression OR profile OR profiling) in the title/abstract. The search strategy in detail is shown in Table 1. Two authors (NS and ZZ) will independently search the databases, and all disagreements will be resolved by consulting corresponding authors (ZG and JW). In order to identify other relevant studies, references of all retrieved articles will be further scrutinized to ensure all potentially eligible studies, including reference lists of primary studies, reviews and key journals.

**Table 1:**
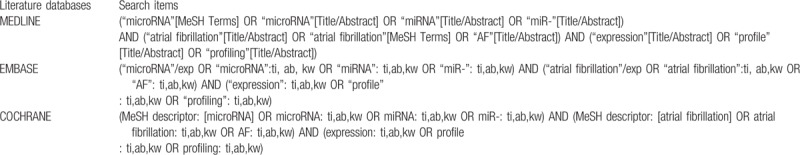
Search strategy used in this study.

### 2.2 Study selection and outcomes

Studies are eligible if the clinical diagnostic criteria are recommended according to reference standards of AHA/ACC/HRS guideline.^[38]^ The clinical evaluation in patients with AF includes: presence and nature of symptoms associated with AF; atrial arrhythmias; difficultly controlled ventricular rate; wide-QRS-complex tachycardia, etc. Due to the rapid update of clinical guidelines, the criteria of reference standard in the primary studies will be adopted. For those studies performed in animal's model, we will also collect information about the model analyzed. All observational studies including cross-sectional, case-control, and cohort will be eligible for inclusion if they meet the following criteria: published original studies that analyzed miRNAs expression using AF and non-AF samples for comparison; miRNA expression studies on patients with AF or animal models of AF; used miRNA microarray or quantitative real-time Polymerase Chain Reaction (PCR) (qRT-PCR) experiments; cut-off criteria of differentially expressed miRNAs and sample sizes are reported. Cell studies, review articles, meeting abstracts, case reports, expert opinions, editorials, and letters without original data will be all excluded. Two authors (NS and ZZ) will independently assess all the studies for determining eligibility, with discrepancies and uncertainties being resolved by corresponding authors (ZG and JW).

### 2.3 Data collection and analysis

The data of inclusion studies will be extracted and recorded independently by 2 reviewers according to the inclusion and exclusion criteria of this systematic review. The following data will be extracted: authors, publication year, ethnicity, clinical characteristics of the patient, miRNA detecting methods, sample types, species, sample size, cut-off criteria of differentially expressed miRNAs, the directory of dysregulated miRNAs, and the total number of differentially expressed miRNA. For same data reported in multiple studies, we will classify them as one study. Disagreements will be resolved by consensus and consultation with a third author (ZG).

### 2.4 Assessment of methodological quality

The quality of studies will be independently assessed by 2 investigators according to the revised version of Quality Assessment of Diagnostic Accuracy Studies-2 (QUADAS-2) which is supported by 7 questions.^[39]^ Each question will be answered with “yes,” “no” or “unclear.” “Yes,” “unclear,” and “no” are given for a score of 1, 0.5, and 0, respectively. Quality scores range from 0 to 7, and the higher score is representative for better quality. The studies will be classified as “high quality” (scores 6–7), “moderate” (scores 4–5), and “low” (scores <3). In addition, the articles will be checked whether they are in accordance to Minimum Information about a Microarray Experiment (MIAME) guideline version 2.0^[40]^ or Minimum Information for Publication of Quantitative Real-time PCR Experiments (MIQE) guideline.^[41]^ Only articles consistent with the above guidelines will be included in this study. Any disagreements will be resolved by consensus.

### 2.5 Heterogeneity assessment

The sources of heterogeneity may derive from subgroup analysis including species (human or animal), miRNA detecting methods (microarray or qRT-PCR), sample types (blood or tissue), population characteristics (sex, age, or disease types), and ethnicities (Asian or non-Asian). The degree of heterogeneity will be investigated by heterogeneity *I*^2^, and *I*^2^ value <50% is recognized as a considerable heterogeneity. Conversely, *I*^2^ value >50% indicates the existence of heterogeneity.^[42]^

### 2.6 Assessment of reporting bias

The potential publication bias will be explored by using visual inspection of Funnel plots, as well as quantitative Begg test and Egger test.^[43]^

### 2.7 Statistical analysis and data synthesis

This systematic review and meta-analysis will be conducted using the Stata (version13, Statacorp, College Station, TX) with random-effects models. The results will be presented as log_10_ odds ratios (logORs) with 95% confidence intervals (CIs) based on the numbers of dysregulation events in AF and non-AF samples. Compared with non-AF group, logOR values higher than 1 is considered as miRNA up-regulation and lower than 1 as miRNA down-regulation. A *P* value of <.05 will be recognized as statistically significant. Subgroup analysis will be conducted according to species, detecting methods, sample types, and ethnicities. Sensitivity analysis will be performed to detect the robustness of the findings by excluding one study by turns and examining the influence of each single study on the pooled logORs value.

### 2.8 Bioinformatics prediction and analysis of miRNA's target genes

To explore biomatic functions of miRNAs in AF, consistently dysregulated miRNAs identified from our meta-analysis will be extracted to predict their targets and confirm potentially biological pathways by employing further bioinformatics analysis. To search for miRNA targets, we will apply target prediction algorithms for selected miRNA sequences using miRWalk 2.0, which includes miRTarBase, miRDB, and TargetScan. The compilation of miRNA-target interactions will be based on target genes predicted by at least 2 of above tools for further analyses. Next, we will perform functional enrichment analysis of miRNAs target genes of Kyoto Encyclopedia of Genes and Genomes (KEGG) Pathway and GO analysis, significance for KEGG pathways and GO enrichment will be estimated with DAVID database. It is considered strongly enriched for the genes targeted by selected miRNAs with *P* value <.05.

## 3 Discussion

The miRNAs dysregulation have been considered causal in many diseases, several miRNA-targeted therapeutics are approaching clinical stage including a mimic of miR-34 as a tumor suppressor in phase I clinical trials,^[44]^ and anti-miRs targeted miR-122 in phase II trials for treating hepatitis.^[45]^ Recent studies have indicated that miRNAs are involved in regulating physiological functions such as electrical and structural remodeling of cardiac tissue.^[46,47]^ Evidence has supported that miRNAs are associated with development, regulation, and treatment of AF.^[48,49]^ Nevertheless, among large number of differentially expressed miRNAs in AF, only a small portion can be valuable for therapeutic targets. Moreover, these differently expressed miRNAs were detected in different detecting methods, tissue, and species. Therefore, it is necessary to acquire representative and credible miRNAs as value biomarkers for AF. This systemic review and meta-analysis will declare a heretofore unreported differentially expression profile of miRNAs in AF.

Based on a variety of inconsistent factors, this systematic review will identify the potential miRNA biomarkers in AF. Meanwhile, the bioinformatic analysis will be conducted to predict target genes of dysregulated miRNAs and to identify potential function in the development of AF.

## 4 Conclusions

The results will identify potential biomarkers for AF, and provide effective therapeutic candidates of miRNA target therapy for AF.

### 4.1 Strengths and limitations of this study

To our best knowledge, this is the first study to summary all dysregulated miRNAs and to evaluate the valuable biomarkers for AF in the form of systematic review and meta-analysis. Furthermore, this study will perform bioinformatics analysis to predict the target genes of dysregulated miRNAs in AF and conduct functional enrichment analysis of miRNA target genes. The included studies including cross-sectional, case-control and cohort design in this systematic review, and studies emphases on molecular functions are required to validate our findings. Since the study mainly focuses on the systematic review of human and animal model studies, cell studies are all excluded.

Reporting bias may exist due to only English articles searched in 4 databases, and some studies may be missed. However, this is a necessary step to ensure that the results of this study are specific, currently relevant, and accurately replicable.

## Author contributions

**Conceptualization:** Nan-Nan Shen, Zai-Li Zhang, Zheng Li, Chi Zhang, Hao Li, Jia-Liang Wang, Jun Wang, Zhichun Gu.
